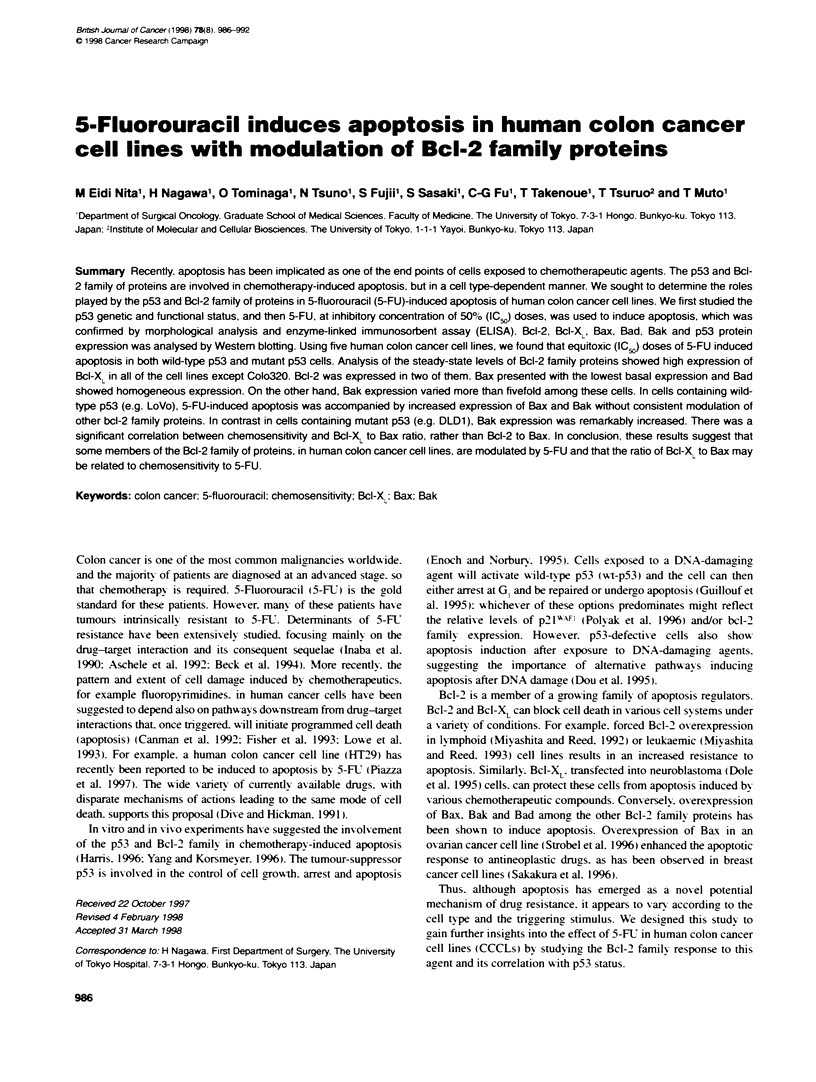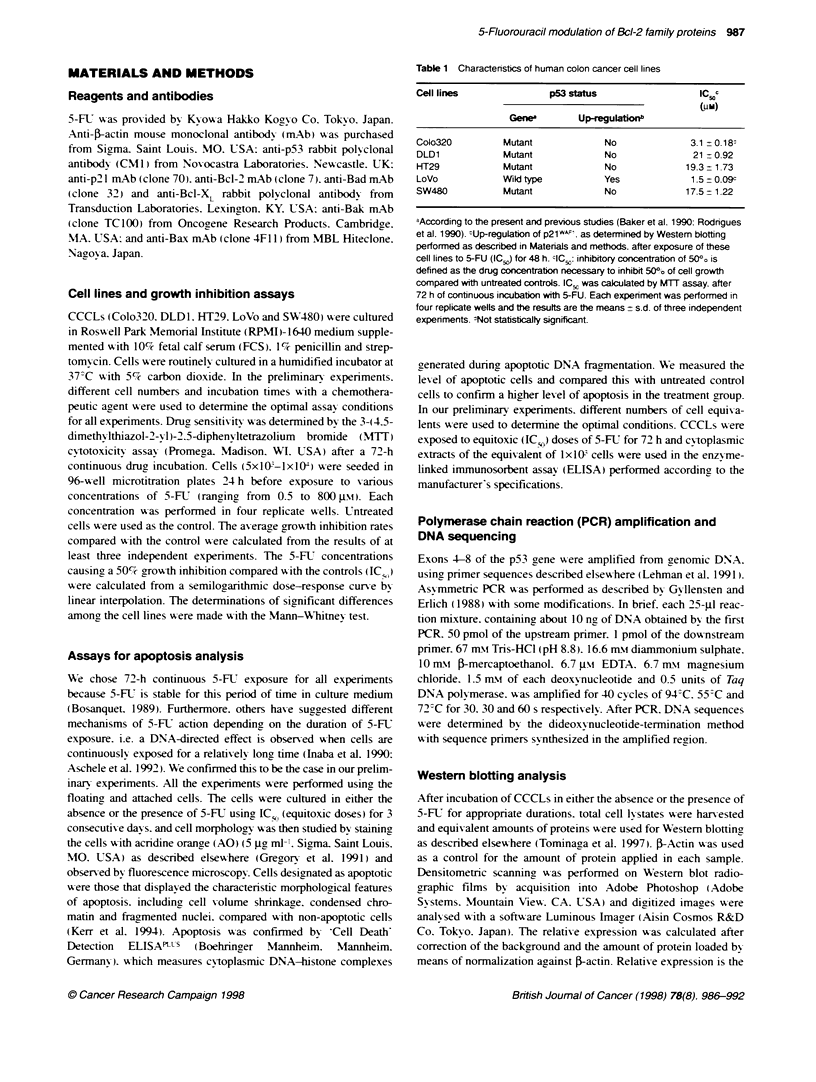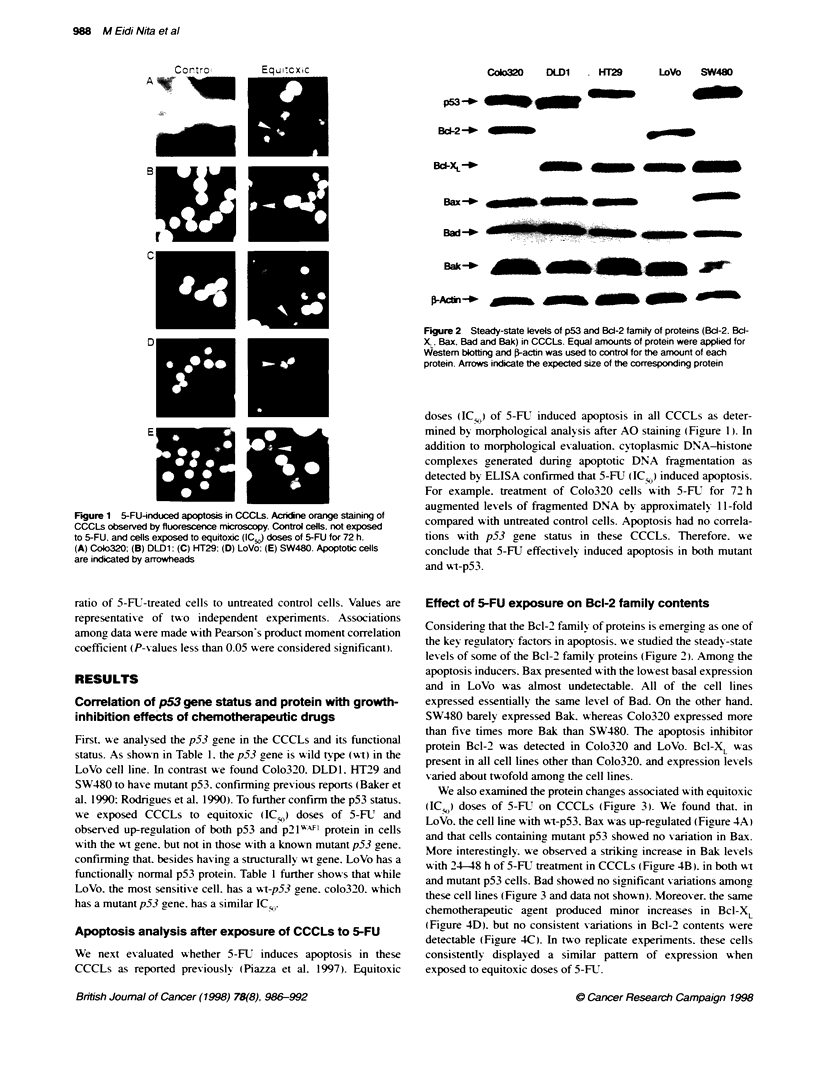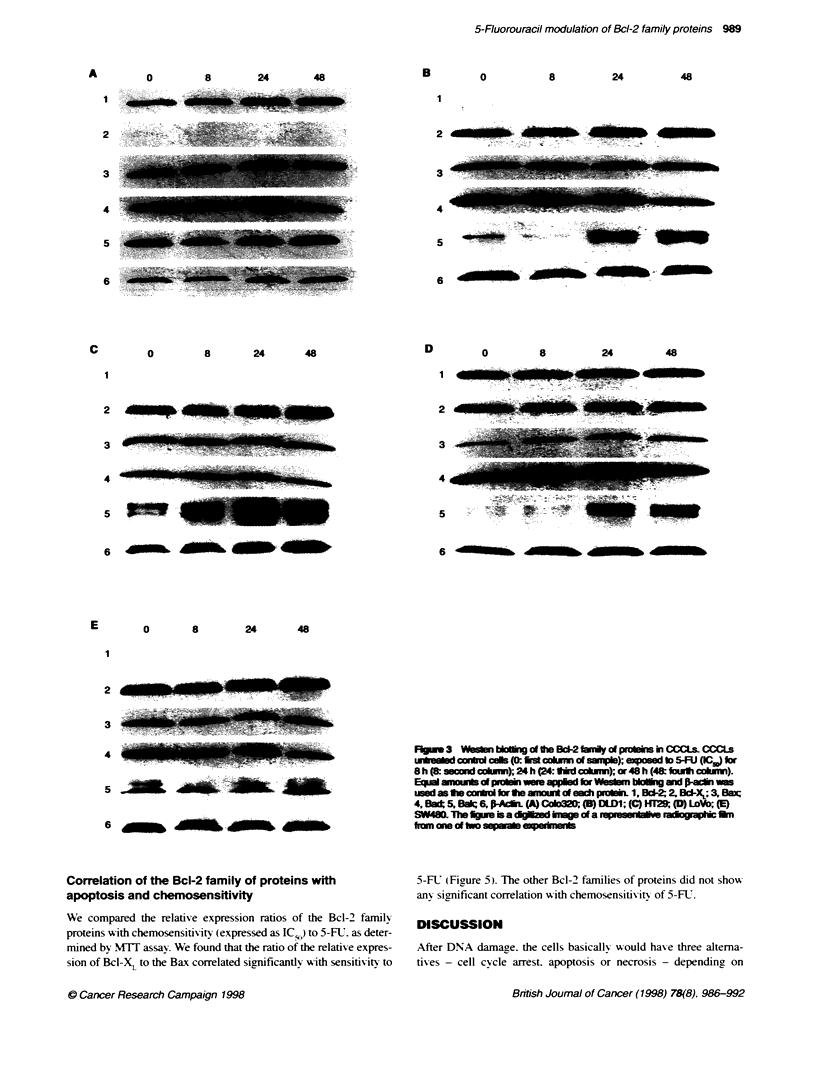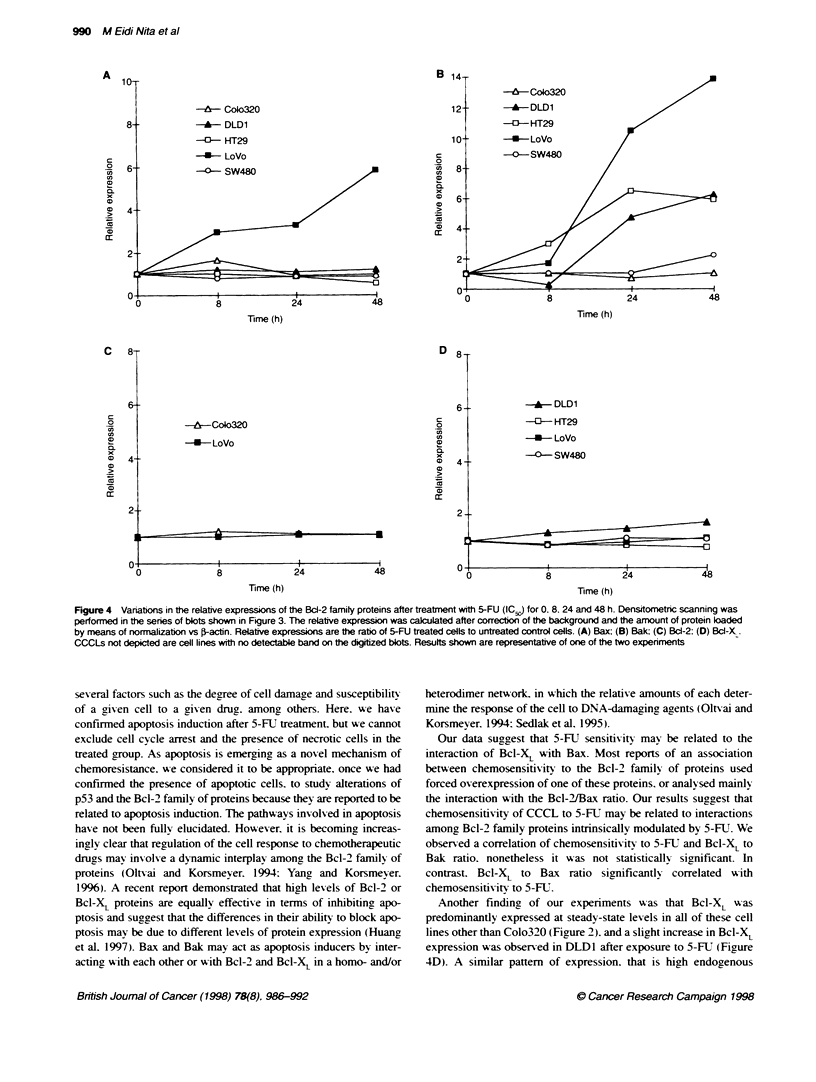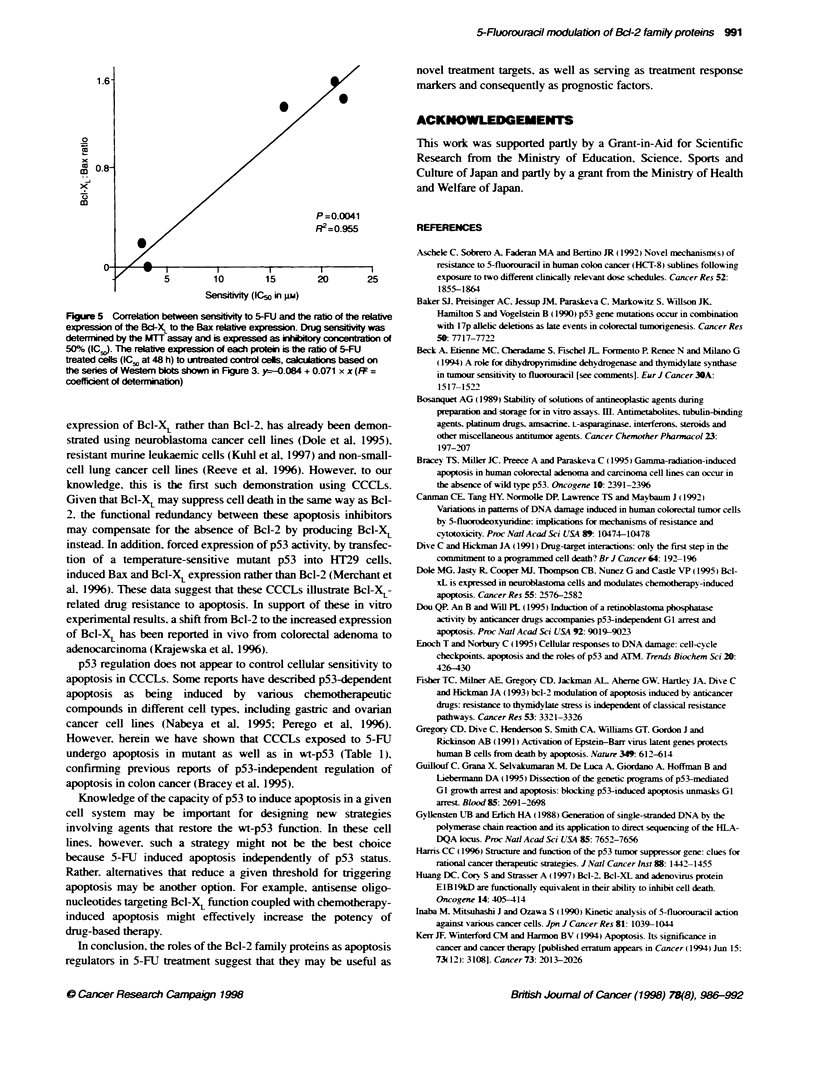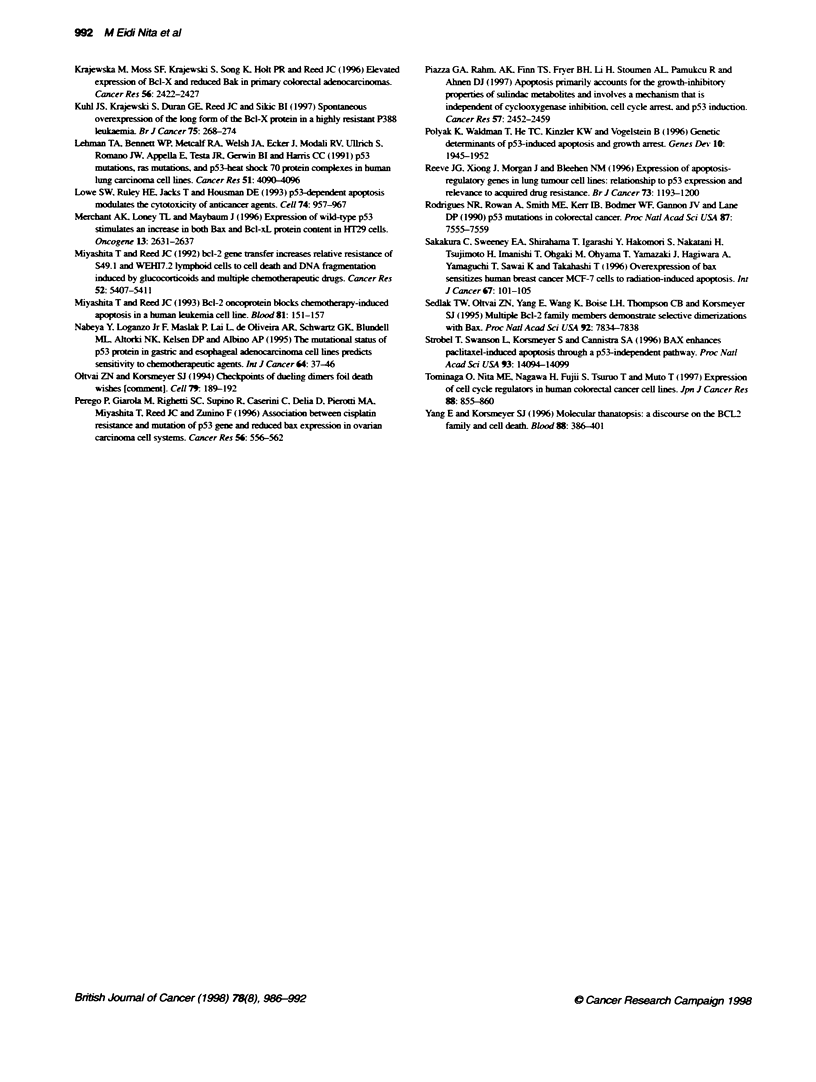# 5-Fluorouracil induces apoptosis in human colon cancer cell lines with modulation of Bcl-2 family proteins.

**DOI:** 10.1038/bjc.1998.617

**Published:** 1998-10

**Authors:** M. E. Nita, H. Nagawa, O. Tominaga, N. Tsuno, S. Fujii, S. Sasaki, C. G. Fu, T. Takenoue, T. Tsuruo, T. Muto

**Affiliations:** Department of Surgical Oncology, Graduate School of Medical Sciences, Faculty of Medicine, The University of Tokyo, Japan.

## Abstract

**Images:**


British Journal of Cancer (1998) 78(8). 986–992

© 1998 Cancer Research Campaign

### 5-Fluorouracil induces apoptosis in human colon cancer cell lines with modulation of Bcl-2 family proteins

**M Eidi Nita[1], H Nagawa[1], O Tominaga[1], N Tsuno[1], S Fujii[1], S Sasaki[1], C-G Fu[1], T Takenoue[1], T Tsuruo[2] and T Muto[1]**

[1]Department of Surgical Oncology. Graduate School of Medical Sciences. Faculty of Medicine. The University of Tokyo. 7-3-1 Hongo. Bunkyo-ku. Tokyo 113. Japan: [2]Institute of Molecular and Cellular Biosciences. The University of Tokyo. 1-1-1 Yayoi. Bunkyo-ku. Tokyo 113. Japan

**Summary** Recently. apoptosis has been implicated as one of the end points of cells exposed to chemotherapeutic agents. The p53 and Bcl-2 family of proteins are involved in chemotherapy-induced apoptosis. but in a cell type-dependent manner. We sought to determine the roles played by the p53 and Bcl-2 family of proteins in 5-fluorouracil (5-FU)-induced apoptosis of human colon cancer cell lines. We first studied the p53 genetic and functional status. and then 5-FU. at inhibitory concentration of 50% ($IC_{50}$) doses, was used to induce apoptosis. which was confirmed by morphological analysis and enzyme-linked immunosorbent assay (ELISA). Bcl-2, Bcl-$X_L$, Bax, Bad, Bak and p53 protein expression was analysed by Western blotting. Using five human colon cancer cell lines, we found that equitoxic ($IC_{50}$) doses of 5-FU induced apoptosis in both wild-type p53 and mutant p53 cells. Analysis of the steady-state levels of Bcl-2 family proteins showed high expression of Bcl-$X_L$ in all of the cell lines except Colo320. Bcl-2 was expressed in two of them. Bax presented with the lowest basal expression and Bad showed homogeneous expression. On the other hand, Bak expression varied more than fivefold among these cells. In cells containing wild-type p53 (e.g. LoVo), 5-FU-induced apoptosis was accompanied by increased expression of Bax and Bak without consistent modulation of other bcl-2 family proteins. In contrast in cells containing mutant p53 (e.g. DLD1), Bak expression was remarkably increased. There was a significant correlation between chemosensitivity and Bcl-$X_L$ to Bax ratio, rather than Bcl-2 to Bax. In conclusion, these results suggest that some members of the Bcl-2 family of proteins, in human colon cancer cell lines, are modulated by 5-FU and that the ratio of Bcl-$X_L$ to Bax may be related to chemosensitivity to 5-FU.

**Keywords:** colon cancer; 5-fluorouracil; chemosensitivity; Bcl-$X_L$; Bax; Bak

Colon cancer is one of the most common malignancies worldwide. and the majority of patients are diagnosed at an advanced stage. so that chemotherapy is required. 5-Fluorouracil (5-FU) is the gold standard for these patients. However. many of these patients have tumours intrinsically resistant to 5-FU. Determinants of 5-FU resistance have been extensively studied. focusing mainly on the drug–target interaction and its consequent sequelae (Inaba et al. 1990: Aschele et al. 1992: Beck et al. 1994). More recently. the pattern and extent of cell damage induced by chemotherapeutics. for example fluoropyrimidines. in human cancer cells have been suggested to depend also on pathways downstream from drug–target interactions that. once triggered. will initiate programmed cell death (apoptosis) (Canman et al. 1992: Fisher et al. 1993: Lowe et al. 1993). For example. a human colon cancer cell line (HT29) has recently been reported to be induced to apoptosis by 5-FU (Piazza et al. 1997). The wide variety of currently available drugs. with disparate mechanisms of actions leading to the same mode of cell death. supports this proposal (Dive and Hickman. 1991).

In vitro and in vivo experiments have suggested the involvement of the p53 and Bcl-2 family in chemotherapy-induced apoptosis (Harris. 1996: Yang and Korsmeyer. 1996). The tumour-suppressor p53 is involved in the control of cell growth. arrest and apoptosis (Enoch and Norbury. 1995). Cells exposed to a DNA-damaging agent will activate wild-type p53 (wt-p53) and the cell can then either arrest at $G_1$ and be repaired or undergo apoptosis (Guillouf et al. 1995): whichever of these options predominates might reflect the relative levels of $p21^{WAF1}$ (Polyak et al. 1996) and/or bcl-2 family expression. However. p53-defective cells also show apoptosis induction after exposure to DNA-damaging agents. suggesting the importance of alternative pathways inducing apoptosis after DNA damage (Dou et al. 1995).

Bcl-2 is a member of a growing family of apoptosis regulators. Bcl-2 and Bcl-$X_L$ can block cell death in various cell systems under a variety of conditions. For example. forced Bcl-2 overexpression in lymphoid (Miyashita and Reed. 1992) or leukaemic (Miyashita and Reed. 1993) cell lines results in an increased resistance to apoptosis. Similarly. Bcl-$X_L$. transfected into neuroblastoma (Dole et al. 1995) cells. can protect these cells from apoptosis induced by various chemotherapeutic compounds. Conversely. overexpression of Bax. Bak and Bad among the other Bcl-2 family proteins has been shown to induce apoptosis. Overexpression of Bax in an ovarian cancer cell line (Strobel et al. 1996) enhanced the apoptotic response to antineoplastic drugs. as has been observed in breast cancer cell lines (Sakakura et al. 1996).

Thus. although apoptosis has emerged as a novel potential mechanism of drug resistance. it appears to vary according to the cell type and the triggering stimulus. We designed this study to gain further insights into the effect of 5-FU in human colon cancer cell lines (CCCLs) by studying the Bcl-2 family response to this agent and its correlation with p53 status.

*Received 22 October 1997*
*Revised 4 February 1998*
*Accepted 31 March 1998*

*Correspondence to:* H Nagawa. First Department of Surgery. The University of Tokyo Hospital. 7-3-1 Hongo. Bunkyo-ku. Tokyo 113. Japan

### MATERIALS AND METHODS

### Reagents and antibodies

5-FU was provided by Kyowa Hakko Kogyo Co. Tokyo. Japan. Anti-β-actin mouse monoclonal antibody (mAb) was purchased from Sigma. Saint Louis. MO. USA: anti-p53 rabbit polyclonal antibody (CM1) from Novocastra Laboratories. Newcastle. UK: anti-p21 mAb (clone 70). anti-Bcl-2 mAb (clone 7). anti-Bad mAb (clone 32) and anti-Bcl-$X_L$ rabbit polyclonal antibody from Transduction Laboratories. Lexington. KY. USA: anti-Bak mAb (clone TC100) from Oncogene Research Products. Cambridge. MA. USA: and anti-Bax mAb (clone 4F11) from MBL Hiteclone. Nagoya. Japan.

### Cell lines and growth inhibition assays

CCCLs (Colo320. DLD1. HT29. LoVo and SW480) were cultured in Roswell Park Memorial Institute (RPMI)-1640 medium supplemented with 10% fetal calf serum (FCS). 1% penicillin and streptomycin. Cells were routinely cultured in a humidified incubator at 37°C with 5% carbon dioxide. In the preliminary experiments. different cell numbers and incubation times with a chemotherapeutic agent were used to determine the optimal assay conditions for all experiments. Drug sensitivity was determined by the 3-(4.5-dimethylthiazol-2-yl)-2.5-diphenyltetrazolium bromide (MTT) cytotoxicity assay (Promega. Madison. WI. USA) after a 72-h continuous drug incubation. Cells ($5\times10^3$–$1\times10^4$) were seeded in 96-well microtitration plates 24 h before exposure to various concentrations of 5-FU (ranging from 0.5 to 800 μM). Each concentration was performed in four replicate wells. Untreated cells were used as the control. The average growth inhibition rates compared with the control were calculated from the results of at least three independent experiments. The 5-FU concentrations causing a 50% growth inhibition compared with the controls ($IC_{50}$) were calculated from a semilogarithmic dose–response curve by linear interpolation. The determinations of significant differences among the cell lines were made with the Mann–Whitney test.

### Assays for apoptosis analysis

We chose 72-h continuous 5-FU exposure for all experiments because 5-FU is stable for this period of time in culture medium (Bosanquet. 1989). Furthermore. others have suggested different mechanisms of 5-FU action depending on the duration of 5-FU exposure. i.e. a DNA-directed effect is observed when cells are continuously exposed for a relatively long time (Inaba et al. 1990: Aschele et al. 1992). We confirmed this to be the case in our preliminary experiments. All the experiments were performed using the floating and attached cells. The cells were cultured in either the absence or the presence of 5-FU using $IC_{50}$ (equitoxic doses) for 3 consecutive days. and cell morphology was then studied by staining the cells with acridine orange (AO) (5 μg $ml^{-1}$. Sigma. Saint Louis. MO. USA) as described elsewhere (Gregory et al. 1991) and observed by fluorescence microscopy. Cells designated as apoptotic were those that displayed the characteristic morphological features of apoptosis. including cell volume shrinkage. condensed chromatin and fragmented nuclei. compared with non-apoptotic cells (Kerr et al. 1994). Apoptosis was confirmed by 'Cell Death' Detection ELISA$^{PLUS}$ (Boehringer Mannheim. Mannheim. Germany). which measures cytoplasmic DNA–histone complexes generated during apoptotic DNA fragmentation. We measured the level of apoptotic cells and compared this with untreated control cells to confirm a higher level of apoptosis in the treatment group. In our preliminary experiments. different numbers of cell equivalents were used to determine the optimal conditions. CCCLs were exposed to equitoxic ($IC_{50}$) doses of 5-FU for 72 h and cytoplasmic extracts of the equivalent of $1\times10^3$ cells were used in the enzyme-linked immunosorbent assay (ELISA) performed according to the manufacturer's specifications.

**Table 1** Characteristics of human colon cancer cell lines

| Cell lines | p53 status | | $IC_{50}$[c] (μM) |
|---|---|---|---|
| | Gene[a] | Up-regulation[b] | |
| Colo320 | Mutant | No | 3.1 ± 0.18[d] |
| DLD1 | Mutant | No | 21 ± 0.92 |
| HT29 | Mutant | No | 19.3 ± 1.73 |
| LoVo | Wild type | Yes | 1.5 ± 0.09[d] |
| SW480 | Mutant | No | 17.5 ± 1.22 |

[a]According to the present and previous studies (Baker et al. 1990: Rodrigues et al. 1990). [b]Up-regulation of p21$^{WAF1}$. as determined by Western blotting performed as described in Materials and methods. after exposure of these cell lines to 5-FU ($IC_{50}$) for 48 h. [c]$IC_{50}$: inhibitory concentration of 50% is defined as the drug concentration necessary to inhibit 50% of cell growth compared with untreated controls. $IC_{50}$ was calculated by MTT assay. after 72 h of continuous incubation with 5-FU. Each experiment was performed in four replicate wells and the results are the means ± s.d. of three independent experiments. [d]Not statistically significant.

### Polymerase chain reaction (PCR) amplification and DNA sequencing

Exons 4–8 of the p53 gene were amplified from genomic DNA. using primer sequences described elsewhere (Lehman et al. 1991). Asymmetric PCR was performed as described by Gyllensten and Erlich (1988) with some modifications. In brief. each 25-μl reaction mixture. containing about 10 ng of DNA obtained by the first PCR. 50 pmol of the upstream primer. 1 pmol of the downstream primer. 67 mM Tris-HCl (pH 8.8). 16.6 mM diammonium sulphate. 10 mM β-mercaptoethanol. 6.7 μM EDTA. 6.7 mM magnesium chloride. 1.5 mM of each deoxynucleotide and 0.5 units of *Taq* DNA polymerase. was amplified for 40 cycles of 94°C. 55°C and 72°C for 30. 30 and 60 s respectively. After PCR. DNA sequences were determined by the dideoxynucleotide-termination method with sequence primers synthesized in the amplified region.

### Western blotting analysis

After incubation of CCCLs in either the absence or the presence of 5-FU for appropriate durations. total cell lystates were harvested and equivalent amounts of proteins were used for Western blotting as described elsewhere (Tominaga et al. 1997). β-Actin was used as a control for the amount of protein applied in each sample. Densitometric scanning was performed on Western blot radiographic films by acquisition into Adobe Photoshop (Adobe Systems. Mountain View. CA. USA) and digitized images were analysed with a software Luminous Imager (Aisin Cosmos R&D Co. Tokyo. Japan). The relative expression was calculated after correction of the background and the amount of protein loaded by means of normalization against β-actin. Relative expression is the

© Cancer Research Campaign 1998

**Figure 1** 5-FU-induced apoptosis in CCCLs. Acridine orange staining of CCCLs observed by fluorescence microscopy. Control cells, not exposed to 5-FU, and cells exposed to equitoxic ($IC_{50}$) doses of 5-FU for 72 h. (**A**) Colo320; (**B**) DLD1; (**C**) HT29; (**D**) LoVo; (**E**) SW480. Apoptotic cells are indicated by arrowheads

**Figure 2** Steady-state levels of p53 and Bcl-2 family of proteins (Bcl-2, Bcl-$X_L$, Bax, Bad and Bak) in CCCLs. Equal amounts of protein were applied for Western blotting and β-actin was used to control for the amount of each protein. Arrows indicate the expected size of the corresponding protein ratio of 5-FU-treated cells to untreated control cells. Values are representative of two independent experiments. Associations among data were made with Pearson's product moment correlation coefficient (*P*-values less than 0.05 were considered significant).

### RESULTS

### Correlation of *p53* gene status and protein with growth-inhibition effects of chemotherapeutic drugs

First, we analysed the *p53* gene in the CCCLs and its functional status. As shown in Table 1, the *p53* gene is wild type (wt) in the LoVo cell line. In contrast we found Colo320, DLD1, HT29 and SW480 to have mutant p53, confirming previous reports (Baker et al, 1990; Rodrigues et al, 1990). To further confirm the p53 status, we exposed CCCLs to equitoxic ($IC_{50}$) doses of 5-FU and observed up-regulation of both p53 and $p21^{WAF1}$ protein in cells with the wt gene, but not in those with a known mutant *p53* gene, confirming that, besides having a structurally wt gene, LoVo has a functionally normal p53 protein. Table 1 further shows that while LoVo, the most sensitive cell, has a wt-*p53* gene, colo320, which has a mutant *p53* gene, has a similar $IC_{50}$.

### Apoptosis analysis after exposure of CCCLs to 5-FU

We next evaluated whether 5-FU induces apoptosis in these CCCLs as reported previously (Piazza et al, 1997). Equitoxic doses ($IC_{50}$) of 5-FU induced apoptosis in all CCCLs as determined by morphological analysis after AO staining (Figure 1). In addition to morphological evaluation, cytoplasmic DNA–histone complexes generated during apoptotic DNA fragmentation as detected by ELISA confirmed that 5-FU ($IC_{50}$) induced apoptosis. For example, treatment of Colo320 cells with 5-FU for 72 h augmented levels of fragmented DNA by approximately 11-fold compared with untreated control cells. Apoptosis had no correlations with *p53* gene status in these CCCLs. Therefore, we conclude that 5-FU effectively induced apoptosis in both mutant and wt-p53.

### Effect of 5-FU exposure on Bcl-2 family contents

Considering that the Bcl-2 family of proteins is emerging as one of the key regulatory factors in apoptosis, we studied the steady-state levels of some of the Bcl-2 family proteins (Figure 2). Among the apoptosis inducers, Bax presented with the lowest basal expression and in LoVo was almost undetectable. All of the cell lines expressed essentially the same level of Bad. On the other hand, SW480 barely expressed Bak, whereas Colo320 expressed more than five times more Bak than SW480. The apoptosis inhibitor protein Bcl-2 was detected in Colo320 and LoVo. Bcl-$X_L$ was present in all cell lines other than Colo320, and expression levels varied about twofold among the cell lines.

We also examined the protein changes associated with equitoxic ($IC_{50}$) doses of 5-FU on CCCLs (Figure 3). We found that, in LoVo, the cell line with wt-p53, Bax was up-regulated (Figure 4A) and that cells containing mutant p53 showed no variation in Bax. More interestingly, we observed a striking increase in Bak levels with 24–48 h of 5-FU treatment in CCCLs (Figure 4B), in both wt and mutant p53 cells. Bad showed no significant variations among these cell lines (Figure 3 and data not shown). Moreover, the same chemotherapeutic agent produced minor increases in Bcl-$X_L$ (Figure 4D), but no consistent variations in Bcl-2 contents were detectable (Figure 4C). In two replicate experiments, these cells consistently displayed a similar pattern of expression when exposed to equitoxic doses of 5-FU.

© Cancer Research Campaign 1998

### Correlation of the Bcl-2 family of proteins with apoptosis and chemosensitivity

We compared the relative expression ratios of the Bcl-2 family proteins with chemosensitivity (expressed as $IC_{50}$) to 5-FU, as determined by MTT assay. We found that the ratio of the relative expression of Bcl-$X_L$ to the Bax correlated significantly with sensitivity to 5-FU (Figure 5). The other Bcl-2 families of proteins did not show any significant correlation with chemosensitivity of 5-FU.

### DISCUSSION

After DNA damage, the cells basically would have three alternatives – cell cycle arrest, apoptosis or necrosis – depending on

© Cancer Research Campaign 1998 

**Figure 4** Variations in the relative expressions of the Bcl-2 family proteins after treatment with 5-FU ($IC_{50}$) for 0, 8, 24 and 48 h. Densitometric scanning was performed in the series of blots shown in Figure 3. The relative expression was calculated after correction of the background and the amount of protein loaded by means of normalization vs β-actin. Relative expressions are the ratio of 5-FU treated cells to untreated control cells. (**A**) Bax; (**B**) Bak; (**C**) Bcl-2; (**D**) Bcl-$X_L$. CCCLs not depicted are cell lines with no detectable band on the digitized blots. Results shown are representative of one of the two experiments several factors such as the degree of cell damage and susceptibility of a given cell to a given drug, among others. Here, we have confirmed apoptosis induction after 5-FU treatment, but we cannot exclude cell cycle arrest and the presence of necrotic cells in the treated group. As apoptosis is emerging as a novel mechanism of chemoresistance, we considered it to be appropriate, once we had confirmed the presence of apoptotic cells, to study alterations of p53 and the Bcl-2 family of proteins because they are reported to be related to apoptosis induction. The pathways involved in apoptosis have not been fully elucidated. However, it is becoming increasingly clear that regulation of the cell response to chemotherapeutic drugs may involve a dynamic interplay among the Bcl-2 family of proteins (Oltvai and Korsmeyer, 1994; Yang and Korsmeyer, 1996). A recent report demonstrated that high levels of Bcl-2 or Bcl-$X_L$ proteins are equally effective in terms of inhibiting apoptosis and suggest that the differences in their ability to block apoptosis may be due to different levels of protein expression (Huang et al, 1997). Bax and Bak may act as apoptosis inducers by interacting with each other or with Bcl-2 and Bcl-$X_L$ in a homo- and/or heterodimer network, in which the relative amounts of each determine the response of the cell to DNA-damaging agents (Oltvai and Korsmeyer, 1994; Sedlak et al, 1995).

Our data suggest that 5-FU sensitivity may be related to the interaction of Bcl-$X_L$ with Bax. Most reports of an association between chemosensitivity to the Bcl-2 family of proteins used forced overexpression of one of these proteins, or analysed mainly the interaction with the Bcl-2/Bax ratio. Our results suggest that chemosensitivity of CCCL to 5-FU may be related to interactions among Bcl-2 family proteins intrinsically modulated by 5-FU. We observed a correlation of chemosensitivity to 5-FU and Bcl-$X_L$ to Bak ratio, nonetheless it was not statistically significant. In contrast, Bcl-$X_L$ to Bax ratio significantly correlated with chemosensitivity to 5-FU.

Another finding of our experiments was that Bcl-$X_L$ was predominantly expressed at steady-state levels in all of these cell lines other than Colo320 (Figure 2), and a slight increase in Bcl-$X_L$ expression was observed in DLD1 after exposure to 5-FU (Figure 4D). A similar pattern of expression, that is high endogenous

© Cancer Research Campaign 1998

**Figure 5** Correlation between sensitivity to 5-FU and the ratio of the relative expression of the Bcl-$X_L$ to the Bax relative expression. Drug sensitivity was determined by the MTT assay and is expressed as inhibitory concentration of 50% ($IC_{50}$). The relative expression of each protein is the ratio of 5-FU treated cells ($IC_{50}$ at 48 h) to untreated control cells, calculations based on the series of Western blots shown in Figure 3. $y=-0.084 + 0.071 \times x$ ($R^2$ = coefficient of determination)

expression of Bcl-$X_L$ rather than Bcl-2, has already been demonstrated using neuroblastoma cancer cell lines (Dole et al, 1995), resistant murine leukaemic cells (Kuhl et al, 1997) and non-small-cell lung cancer cell lines (Reeve et al, 1996). However, to our knowledge, this is the first such demonstration using CCCLs. Given that Bcl-$X_L$ may suppress cell death in the same way as Bcl-2, the functional redundancy between these apoptosis inhibitors may compensate for the absence of Bcl-2 by producing Bcl-$X_L$ instead. In addition, forced expression of p53 activity, by transfection of a temperature-sensitive mutant p53 into HT29 cells, induced Bax and Bcl-$X_L$ expression rather than Bcl-2 (Merchant et al, 1996). These data suggest that these CCCLs illustrate Bcl-$X_L$-related drug resistance to apoptosis. In support of these in vitro experimental results, a shift from Bcl-2 to the increased expression of Bcl-$X_L$ has been reported in vivo from colorectal adenoma to adenocarcinoma (Krajewska et al, 1996).

p53 regulation does not appear to control cellular sensitivity to apoptosis in CCCLs. Some reports have described p53-dependent apoptosis as being induced by various chemotherapeutic compounds in different cell types, including gastric and ovarian cancer cell lines (Nabeya et al, 1995; Perego et al, 1996). However, herein we have shown that CCCLs exposed to 5-FU undergo apoptosis in mutant as well as in wt-p53 (Table 1), confirming previous reports of p53-independent regulation of apoptosis in colon cancer (Bracey et al, 1995).

Knowledge of the capacity of p53 to induce apoptosis in a given cell system may be important for designing new strategies involving agents that restore the wt-p53 function. In these cell lines, however, such a strategy might not be the best choice because 5-FU induced apoptosis independently of p53 status. Rather, alternatives that reduce a given threshold for triggering apoptosis may be another option. For example, antisense oligonucleotides targeting Bcl-$X_L$ function coupled with chemotherapy-induced apoptosis might effectively increase the potency of drug-based therapy.

In conclusion, the roles of the Bcl-2 family proteins as apoptosis regulators in 5-FU treatment suggest that they may be useful as novel treatment targets, as well as serving as treatment response markers and consequently as prognostic factors.

### ACKNOWLEDGEMENTS

This work was supported partly by a Grant-in-Aid for Scientific Research from the Ministry of Education, Science, Sports and Culture of Japan and partly by a grant from the Ministry of Health and Welfare of Japan.

### REFERENCES

Aschele C, Sobrero A, Faderan MA and Bertino JR (1992) Novel mechanism(s) of resistance to 5-fluorouracil in human colon cancer (HCT-8) sublines following exposure to two different clinically relevant dose schedules. *Cancer Res* **52**: 1855–1864

Baker SJ, Preisinger AC, Jessup JM, Paraskeva C, Markowitz S, Willson JK, Hamilton S and Vogelstein B (1990) p53 gene mutations occur in combination with 17p allelic deletions as late events in colorectal tumorigenesis. *Cancer Res* **50**: 7717–7722

Beck A, Etienne MC, Cheradame S, Fischel JL, Formento P, Renee N and Milano G (1994) A role for dihydropyrimidine dehydrogenase and thymidylate synthase in tumour sensitivity to fluorouracil [see comments]. *Eur J Cancer* **30A**: 1517–1522

Bosanquet AG (1989) Stability of solutions of antineoplastic agents during preparation and storage for in vitro assays. III. Antimetabolites, tubulin-binding agents, platinum drugs, amsacrine, L-asparaginase, interferons, steroids and other miscellaneous antitumor agents. *Cancer Chemother Pharmacol* **23**: 197–207

Bracey TS, Miller JC, Preece A and Paraskeva C (1995) Gamma-radiation-induced apoptosis in human colorectal adenoma and carcinoma cell lines can occur in the absence of wild type p53. *Oncogene* **10**: 2391–2396

Canman CE, Tang HY, Normolle DP, Lawrence TS and Maybaum J (1992) Variations in patterns of DNA damage induced in human colorectal tumor cells by 5-fluorodeoxyuridine: implications for mechanisms of resistance and cytotoxicity. *Proc Natl Acad Sci USA* **89**: 10474–10478

Dive C and Hickman JA (1991) Drug-target interactions: only the first step in the commitment to a programmed cell death? *Br J Cancer* **64**: 192–196

Dole MG, Jasty R, Cooper MJ, Thompson CB, Nunez G and Castle VP (1995) Bcl-xL is expressed in neuroblastoma cells and modulates chemotherapy-induced apoptosis. *Cancer Res* **55**: 2576–2582

Dou QP, An B and Will PL (1995) Induction of a retinoblastoma phosphatase activity by anticancer drugs accompanies p53-independent G1 arrest and apoptosis. *Proc Natl Acad Sci USA* **92**: 9019–9023

Enoch T and Norbury C (1995) Cellular responses to DNA damage: cell-cycle checkpoints, apoptosis and the roles of p53 and ATM. *Trends Biochem Sci* **20**: 426–430

Fisher TC, Milner AE, Gregory CD, Jackman AL, Aherne GW, Hartley JA, Dive C and Hickman JA (1993) bcl-2 modulation of apoptosis induced by anticancer drugs: resistance to thymidylate stress is independent of classical resistance pathways. *Cancer Res* **53**: 3321–3326

Gregory CD, Dive C, Henderson S, Smith CA, Williams GT, Gordon J and Rickinson AB (1991) Activation of Epstein–Barr virus latent genes protects human B cells from death by apoptosis. *Nature* **349**: 612–614

Guillouf C, Grana X, Selvakumaran M, De Luca A, Giordano A, Hoffman B and Liebermann DA (1995) Dissection of the genetic programs of p53-mediated G1 growth arrest and apoptosis: blocking p53-induced apoptosis unmasks G1 arrest. *Blood* **85**: 2691–2698

Gyllensten UB and Erlich HA (1988) Generation of single-stranded DNA by the polymerase chain reaction and its application to direct sequencing of the HLA-DQA locus. *Proc Natl Acad Sci USA* **85**: 7652–7656

Harris CC (1996) Structure and function of the p53 tumor suppressor gene: clues for rational cancer therapeutic strategies. *J Natl Cancer Inst* **88**: 1442–1455

Huang DC, Cory S and Strasser A (1997) Bcl-2, Bcl-XL and adenovirus protein E1B19kD are functionally equivalent in their ability to inhibit cell death. *Oncogene* **14**: 405–414

Inaba M, Mitsuhashi J and Ozawa S (1990) Kinetic analysis of 5-fluorouracil action against various cancer cells. *Jpn J Cancer Res* **81**: 1039–1044

Kerr JF, Winterford CM and Harmon BV (1994) Apoptosis. Its significance in cancer and cancer therapy [published erratum appears in *Cancer* (1994) Jun 15; 73(12): 3108]. *Cancer* **73**: 2013–2026

© Cancer Research Campaign 1998 

Krajewska M, Moss SF, Krajewski S, Song K, Holt PR and Reed JC (1996) Elevated expression of Bcl-X and reduced Bak in primary colorectal adenocarcinomas. *Cancer Res* **56**: 2422–2427

Kuhl JS, Krajewski S, Duran GE, Reed JC and Sikic BI (1997) Spontaneous overexpression of the long form of the Bcl-X protein in a highly resistant P388 leukaemia. *Br J Cancer* **75**: 268–274

Lehman TA, Bennett WP, Metcalf RA, Welsh JA, Ecker J, Modali RV, Ullrich S, Romano JW, Appella E, Testa JR, Gerwin BI and Harris CC (1991) p53 mutations, ras mutations, and p53-heat shock 70 protein complexes in human lung carcinoma cell lines. *Cancer Res* **51**: 4090–4096

Lowe SW, Ruley HE, Jacks T and Housman DE (1993) p53-dependent apoptosis modulates the cytotoxicity of anticancer agents. *Cell* **74**: 957–967

Merchant AK, Loney TL and Maybaum J (1996) Expression of wild-type p53 stimulates an increase in both Bax and Bcl-xL protein content in HT29 cells. *Oncogene* **13**: 2631–2637

Miyashita T and Reed JC (1992) bcl-2 gene transfer increases relative resistance of S49.1 and WEHI7.2 lymphoid cells to cell death and DNA fragmentation induced by glucocorticoids and multiple chemotherapeutic drugs. *Cancer Res* **52**: 5407–5411

Miyashita T and Reed JC (1993) Bcl-2 oncoprotein blocks chemotherapy-induced apoptosis in a human leukemia cell line. *Blood* **81**: 151–157

Nabeya Y, Loganzo Jr F, Maslak P, Lai L, de Oliveira AR, Schwartz GK, Blundell ML, Altorki NK, Kelsen DP and Albino AP (1995) The mutational status of p53 protein in gastric and esophageal adenocarcinoma cell lines predicts sensitivity to chemotherapeutic agents. *Int J Cancer* **64**: 37–46

Oltvai ZN and Korsmeyer SJ (1994) Checkpoints of dueling dimers foil death wishes [comment]. *Cell* **79**: 189–192

Perego P, Giarola M, Righetti SC, Supino R, Caserini C, Delia D, Pierotti MA, Miyashita T, Reed JC and Zunino F (1996) Association between cisplatin resistance and mutation of p53 gene and reduced bax expression in ovarian carcinoma cell systems. *Cancer Res* **56**: 556–562

Piazza GA, Rahm, AK, Finn TS, Fryer BH, Li H, Stoumen AL, Pamukcu R and Ahnen DJ (1997) Apoptosis primarily accounts for the growth-inhibitory properties of sulindac metabolites and involves a mechanism that is independent of cyclooxygenase inhibition, cell cycle arrest, and p53 induction. *Cancer Res* **57**: 2452–2459

Polyak K, Waldman T, He TC, Kinzler KW and Vogelstein B (1996) Genetic determinants of p53-induced apoptosis and growth arrest. *Genes Dev* **10**: 1945–1952

Reeve JG, Xiong J, Morgan J and Bleehen NM (1996) Expression of apoptosis-regulatory genes in lung tumour cell lines: relationship to p53 expression and relevance to acquired drug resistance. *Br J Cancer* **73**: 1193–1200

Rodrigues NR, Rowan A, Smith ME, Kerr IB, Bodmer WF, Gannon JV and Lane DP (1990) p53 mutations in colorectal cancer. *Proc Natl Acad Sci USA* **87**: 7555–7559

Sakakura C, Sweeney EA, Shirahama T, Igarashi Y, Hakomori S, Nakatani H, Tsujimoto H, Imanishi T, Ohgaki M, Ohyama T, Yamazaki J, Hagiwara A, Yamaguchi T, Sawai K and Takahashi T (1996) Overexpression of bax sensitizes human breast cancer MCF-7 cells to radiation-induced apoptosis. *Int J Cancer* **67**: 101–105

Sedlak TW, Oltvai ZN, Yang E, Wang K, Boise LH, Thompson CB and Korsmeyer SJ (1995) Multiple Bcl-2 family members demonstrate selective dimerizations with Bax. *Proc Natl Acad Sci USA* **92**: 7834–7838

Strobel T, Swanson L, Korsmeyer S and Cannistra SA (1996) BAX enhances paclitaxel-induced apoptosis through a p53-independent pathway. *Proc Natl Acad Sci USA* **93**: 14094–14099

Tominaga O, Nita ME, Nagawa H, Fujii S, Tsuruo T and Muto T (1997) Expression of cell cycle regulators in human colorectal cancer cell lines. *Jpn J Cancer Res* **88**: 855–860

Yang E and Korsmeyer SJ (1996) Molecular thanatopsis: a discourse on the BCL2 family and cell death. *Blood* **88**: 386–401

© Cancer Research Campaign 1998